# From Crashing Waves to Crashing Sodium: A Rare Case of Nearly Asymptomatic Severe Hyponatremia

**DOI:** 10.7759/cureus.41474

**Published:** 2023-07-06

**Authors:** Fawaz Hussain, Natalie E Ebeling-Koning, Joshua Carlson, Gillian A Beauchamp

**Affiliations:** 1 Internal Medicine, University of South Florida Morsani College of Medicine, Tampa, USA; 2 Department of Emergency and Hospital Medicine, Lehigh Valley Health Network, Allentown, USA; 3 Department of Pulmonary and Critical Care Medicine, Lehigh Valley Health Network, Allentown, USA

**Keywords:** severe hyponatremia, desmopressin, siadh, case report, sodium, asymptomatic

## Abstract

Hyponatremia refers to an abnormally low serum sodium level, and it is the most common electrolyte disorder encountered in the clinical setting. Despite its prevalence, hyponatremia can be challenging to clinically identify in some cases due to non-specific symptom presentation. In this case report, we illustrate the rare clinical course of a nearly asymptomatic patient with severe hyponatremia and discuss potential explanations for this uncommon presentation.

## Introduction

Hyponatremia is characterized by a serum sodium level under 135 mmol/L and is the most common electrolyte imbalance encountered in the hospital setting [1]. Abrupt changes in volume status, hormones, and medications (e.g., diuretics, antidepressants, and antiepileptics) can contribute to patients developing hyponatremia [2]. Depending on the pathophysiologic severity of the sodium imbalance, it is known to cause a spectrum of neurological, musculoskeletal, and gastrointestinal symptoms that can progress to life-threatening complications [3].

Severe hyponatremia (serum sodium less than 125 mmol/L) has been associated with increased length of hospitalization and significantly higher mortality risk [4]. A large retrospective study conducted by Chawla et al. reported an overall mortality rate of 6.1% in hospitalized patients with hyponatremia - notably higher than the overall mortality rate of 2.3% among patients with a normal serum sodium level. Thus, early diagnosis and appropriate management of hyponatremia are crucial to prevent complications and improve clinical outcomes.

## Case presentation

A 47-year-old male patient with a past medical history of type 2 diabetes mellitus, hypertension, and alcohol use disorder presented to the emergency department (ED) for worsening headache, blurry vision, nausea, and vomiting following a fall four days prior to arrival. The patient had been knocked over by aggressive waves at the beach and experienced head trauma with a brief loss of consciousness. 

On physical examination, the patient was in no acute distress with a Glasgow Coma Scale score of 15. He had a temperature of 97.3°F, blood pressure of 149/89 mmHg, heart rate of 86 beats/min, respiratory rate of 18 breaths/min, and oxygen saturation of 96% on room air. His exam was remarkable for mild generalized musculoskeletal tenderness but was otherwise normal including normal mental status and neurological exam.

The patient's ED evaluation incidentally identified severe hypoosmolar hyponatremia with serum sodium of 99 mmol/L, serum potassium of 2.2 mmol/L, serum magnesium of 1.1 mmol/L, lactate of 3.7 mmol/L, high anion gap of 14, glucose of 212 mg/dL, serum osmolality of 214 mOsm/kg, and urine osmolality of 562 mOsm/kg with adequate urine output and moist mucous membranes. Venous blood gas analysis revealed a pH of 7.62 and pCO_2_ of 35, concerning for respiratory alkalosis masking an underlying metabolic acidosis. CT head without contrast did not show any evidence of intracranial hemorrhage, epidural hematoma, or skull fracture.

The nephrology team was consulted, and the patient was admitted to the intensive care unit (ICU). Intravenous (IV) 3% hypertonic saline and 2 mcg of desmopressin twice daily were administered to gradually correct the serum sodium by 6-8 mmol/L daily. Desmopressin was utilized to prevent a large-volume diuresis and was withheld when appropriate to achieve this target correction rate. The patient was also given potassium chloride and magnesium sulfate intravenously until resolution of his concurrent hypokalemia and hypomagnesemia, respectively. He was placed on strict fluid restriction, and his electrolytes were monitored every four hours. Evaluation by medical toxicology identified early signs of alcohol withdrawal, which was treated with IV phenobarbital. The patient reported heavy alcohol use for several months in addition to ingesting approximately 320 ounces of water daily and restricting food intake. Three months prior, the patient had also begun taking 50 mg of chlorthalidone daily.

Over the next 18 hours, the patient’s serum sodium increased from 99 mmol/L to 104 mmol/L. Despite still being severely hyponatremic, his mental status remained normal. The 3% hypertonic saline and desmopressin were continued for a total of five days for gradual sodium correction until serum sodium was 133 mmol/L, nearly normal.

The patient was in good condition and electrolytes were normal at discharge on the eighth day at the hospital. Chlorthalidone was discontinued from his medication regimen, and he was scheduled for a close outpatient follow-up to reduce the risk of re-admission.

## Discussion

Identifying the clinical signs of mild or moderate hyponatremia can be challenging due to less pronounced symptoms and overlap with other clinical presentations. However, severe hyponatremia typically presents with life-threatening symptoms including seizures, respiratory arrest, and coma [5]. In this unusual case of a patient with a Glasgow Coma Scale of 15 and no cognitive or neurological deficits, serum sodium of less than 100 mmol/L is unexpected. The patient’s presenting symptoms of headache, nausea, and vomiting are non-specific and could be attributed to many causes.

Given the severity of the hyponatremia, it is imperative to avoid rapid overcorrection, no more than 10 mmol/L in 24 hours, due to the risk of osmotic demyelination syndrome (ODS) [6]. Signs concerning ODS typically develop over several days following sodium overcorrection and include quadriparesis, dysphagia, dysarthria, seizures, altered mental status, and coma [7-9]. One multicenter observational study followed 36 patients with ODS over one year and reported that 31% died within one year and another 31% required life-supporting therapies [10].

Regarding this case of severe hypoosmolar euvolemic hyponatremia, the suspected etiology was a combination of the underlying syndrome of inappropriate antidiuretic hormone (SIADH) likely secondary to chlorthalidone use, psychogenic polydipsia, and decreased solute intake from excessive alcohol use. Thiazide diuretics such as chlorthalidone are known to induce hyponatremia by enhancing antidiuretic hormone secretion, increasing urinary sodium excretion, and increasing water intake [11,12].

The treatment plan was tailored to gradually correct the serum sodium by 6-8 mmol/L in the first 24 hours using 3% hypertonic saline along with desmopressin to prevent water losses [13]. Given this patient’s risk of large-volume water diuresis, desmopressin was utilized proactively to minimize free water excretion and limit autocorrection of serum sodium. It is key to restrict fluids and monitor urine output while the patient is on desmopressin; otherwise, unrestricted fluid intake can induce further hyponatremia [14].

## Conclusions

This case report highlights the importance of evaluating the serum sodium level even in the case of non-specific symptoms, as failure to do so can lead to devastating consequences. In addition to ordering a comprehensive lab work-up, conducting a thorough history and physical exam are essential in understanding the underlying etiologies of hyponatremia. Due to the variable presentation of hyponatremia, clinicians must have a high index of suspicion to facilitate prompt diagnosis and effective management. Gradual correction of serum sodium and fluid restriction are vital for reducing the risk of ODS.
